# Association of the Individual and Context Inequalities on the Breastfeeding: A Study from the Sicily Region

**DOI:** 10.3390/ijerph16193514

**Published:** 2019-09-20

**Authors:** Achille Cernigliaro, Sara Palmeri, Alessandra Casuccio, Salvatore Scondotto, Vincenzo Restivo, In Primis Working Group

**Affiliations:** 1 Department of Health Services and Epidemiological Observatory, Regional Health Authority, 90145 Palermo, Italysalvatore.scondotto@regione.sicilia.it (S.S.); 2 Department of Health Promotion, Maternal and Infant Care, Internal Medicine and Medical Specialties, “G. D’Alessandro”, University of Palermo, 90127 Palermo, Italy; sarapalmeri@libero.it (S.P.); vincenzo.restivo@unipa.it (V.R.); vincenzo_restivo83@hotmail.it

**Keywords:** Exclusive breastfeeding, predominant breastfeeding, complementary breastfeeding, individual deprivation, context deprivation, prospective study, Southern Italy, Women’s health

## Abstract

Despite the advantages of breastfeeding being widely recognized, the economic level can have an influence on breastfeeding rates, with rich women breastfeeding longer than poor in high-income countries. In Italy, socio-economic differences affect breastfeeding start and continuation among most deprived people, such as in Southern Italy. The objective of the study was to evaluate the prevalence of the initiation and continuation of exclusive breastfeeding and its association with the levels of socio-economic deprivation in Sicily. A prospective cohort study with a two-phase survey in three breastfeeding detection times was conducted. Overall, 1,055 mothers were recruited with a mean age of 31 years. Breastfeeding decreased from 86% during hospitalization to 69% at the first month and 42% at the sixth month, yet at the same time, exclusive breastfeeding increased from 34% to 38% during hospitalization to the first month and went down to 20.2% at the sixth month. The adjusted multivariate analysis showed no association with individual inequalities. On the other hand, the context inequalities had a significant association with the risk of not following exclusive breastfeeding in the deprived class (odds ratio (OR): 2.08, confidence interval (CI) 95% 1.01–4.27) and in the very deprived class (OR: 1.83, CI 95% 1.00–3.38) at the six-month survey. These results indicate that the context inequalities begin to emerge from the return home of the mother and the child.

## 1. Introduction

Human milk is a complex tissue made up of fundamental elements for the development and health of the child [1]. The benefits of breastfeeding are widely recognized and concern benefits for the child, mother and society. Results of biological and epidemiological studies confirm that failure to breastfeed has important short-term effects on child health [2,3,4], such as increased respiratory and gastrointestinal infections, hospital admissions, sudden infant death syndrome (SIDS), and long-term effects, such as increases in obesity, diabetes, tumors and intelligence quotient reduction [3,5,6,7,8,9]. Failure to breastfeed can also lead to effects on women's health, such us increased breast cancer, ovary cancer, overweight, osteoporosis and postpartum depression [6,10,11,12]. Consequently, unmotivated replacement of breast milk with artificial milk is not beneficial, and it could compromise the health of the child and mother.

The Lives Saved Tool estimates that breastfeeding could potentially prevent more than 800,000 child deaths worldwide each year [13]. This corresponds to 13.8% of the deaths among children under 2 years old [14,15]. In particular, 87% of preventable deaths would have occurred in children under the age of 6 months, due to a combination of high mortality rates and low prevalence of exclusive breastfeeding. In developing countries, a study found that children younger than 6 months who were not breastfed had 4 times higher mortality rates than breastfed children [16]. These results are confirmed by studies among children aged 6–23 months, in which breastfeeding was associated with a 50% reduction in deaths [17]. Furthermore, breastfeeding reduces child mortality rates even in high-income countries among children < 5 years old, although to a lesser extent [18]. Moreover, it is estimated that global breastfeeding rates prevent around 19,464 annual breast cancer deaths and another 22,216 lives a year would be saved through increasing the duration of breastfeeding [19].

The World Health Organization (WHO) and the United Nations Children's Fund (UNICEF) recognize breastfeeding as a fundamental right of the mother and child. Breast milk is widely recommended by the WHO as the exclusive food for the first six months of the child's life and it is complementary up to two years and more, if the mother and child desire it [20]. On the other hand, the marketing of breast-milk substitutes is the most large and competitive infant feeding industry with an amount of 44.8 billion US dollars global sales in 2014. It is estimated that breastfeeding would allow for saving about 302 billion US dollars per year or 0.49% of world gross national income [21]. Furthermore, it is easy to appreciate how breast milk has lower environmental impacts than artificial industrially produced milk: breastfeeding contributes to the development of a healthier planet because its lower ecological footprint, compared to the artificial formula, reduces its use of water, energy, paper and metal [21].

In most countries, exclusive breastfeeding rates are well below 50% [21]. In the USA, recent data indicate that less than 50% of children are exclusively breastfed in the first 3 months and only about 25% are exclusively breastfed for 6 months [22]. In Italy, according to the 2013 estimates of the National Institute of Statistics (Istat), the prevalence of exclusive breastfeeding among 4–5-month-old children is 38.6% [23]. However, even in low-income and middle-income countries, only 37% of children under the age of 6 months are exclusively breastfed [21]. Literature suggests that the economic level can have an influence on breastfeeding rates, with poor women breastfeeding longer than rich women in developing countries, and the opposite in high-income countries [21]. These results suggest that investigate breastfeeding models may help to reduce health gaps among rich and poor children in developing and developed countries.

In Italy, socio-economic and geographical differences affect breastfeeding start and continuation. Indeed, breastfeeding rates decrease among people with lower levels of education and socio-economic status, such as in Southern Italy [23]. In a more recent survey conducted at the Sicilian vaccination centers in 2015 [24], 30.6% of mothers declared to have exclusively breast-fed, 23.1% to have breastfed with formula supplementation and 46.3% fed infants only with formula. In 2017, in Sicily, a prospective cohort study called “In Primis – Primal Health, the first thousand days of our children” was conducted. The In Primis Study, promoted by the Regional Health Authority Department in collaboration with the University of Palermo and the National Institute of Health, aimed to identify the vulnerability factors of mothers and children during pregnancy, birth and puerperium, according to the National and Regional Prevention Plans [25,26,27].

The objective of the study was to measure the prevalence of the initiation and continuation of exclusive breastfeeding in Sicily at birth, one month, and six months of life, stratifying by the levels of socio-economic deprivation.

## 2. Materials and Methods 

### 2.1. Study Design

The In Primis prospective cohort study, with a two-phase survey in three breastfeeding detection times, was conducted:

The first phase consists of a questionnaire administration, within 30 days of birth. In this phase, information was detected concerning two periods: hospital stay and the first month of the child’s life. The questionnaire used in this study was built up by a previous questionnaire already used in Italy [28]. The questionnaire was divided into four sections: pregnancy, birth, postpartum, and personal and socio-demographic characteristics. Each section of the questionnaire referred to multiple variables, such as participation in a pregnancy course for expecting parents, type of birth, organization of birth healthcare services, assistance during hospitalization, smoking habits, age and education.

The second phase was the administration of the questionnaire between the 150th day and the 171st day of postnatal life.

To evaluate the association between breastfeeding and socio-economic inequalities, two socio-economic position (SEP) indexes have been built up: an individual SEP, as an expression of the individual mother's level of deprivation and a context SEP (produced according to municipality of residence), as an expression of deprivation due to context where the mothers live. The individual SEP included some dimensions of disadvantage which were detected through the questionnaire: marital status, cohabitation, education, nationality and monthly economic intake. The sum of different dimension produced a continuous index that was divided into quintiles of distribution: 1—very rich, 2—rich, 3—medium, 4—deprived and 5—very deprived.

The context SEP considered the following disadvantage dimensions: instruction, occupation, housing conditions and families. This was a validated technique at the national level and applied to Sicilian municipalities using the date of the last Italian census of Istat on population and housing (year 2011) [29,30]. The Z transformation methodology was applied to the items, and their sum produced an index that was divided into quintiles of population like the previous SEP: 1—very rich, 2—rich, 3—medium, 4—deprived and 5—very deprived. Before the interview, aims as well as methods used to ensure data confidentiality were explained and informed consent was obtained from all participants. The In Primis project was approved by the Ethics Committee of Palermo 1 in the second session of 2017.

### 2.2. Sample Size

The sample size was determined considering the 2015 Sicilian births cohort (N = 43,187), the breastfeeding prevalence of 50%, a confidence level of 95% and an accuracy of ±4.5%. The sample size was stratified by the amount of newborns among the 9 Sicilian provinces. The sampling methodology used was the systematic one with a step equal to 6. The sample included women aged 18 or over, residing in Sicily who gave birth between March 1 and May 30, 2017 and who had a telephone number. For each of these women defined as "owner", five other women called "substitutes", were used when the “owner” woman was: not on call by multiple calls on different days and times, not available to answer, no knowledge of the Italian language and erroneous telephone number on the list.

### 2.3. Statistical Analysis

A descriptive analysis of the socio-economic characteristics of mothers and other variables associated with breastfeeding was performed. Breastfeeding was evaluated in three detection times, the hospital stay, at 1 and 6 months, according to the definition of the WHO: exclusive breastfeeding (nutrition with only breast milk, including squeezed or donated breast milk, without other foods, liquids or water except drugs, vitamins, minerals or rehydrating solutions), prevalent (nutrition with breast milk with addiction of non-nutritive liquids like water, glucose solution, herbal teas and juices), complementary (breast milk with the addition of any other food, semi-liquid or solid, including milk of other species) and non-breastfeeding (formulated breast milk substitutes or any other food, semi-liquid or solid, including milk of from other species) [20].

To evaluate the risk of deprivation in adherence to the exclusive breastfeeding, for each of the disadvantage levels (in both individual and context SEP) and for each of the three breastfeeding detection times, associations (χ^2^), the trend (χ^2^ trend) and the crude and adjusted risks were estimated with an alfa error equal to 0.05. The odds ratio (OR) of not exclusive breastfeeding and the relative 95% confidence intervals (CIs) were calculated, using a logistic regression model, which included the variables detected through the questionnaire as possible determinants of breastfeeding habit.

## 3. Results

A total of 1055 mothers were recruited between April and July 2017, of which 643 were substitutions (33% of the sample). The mean age of the women was 31 years (range 18–46) and 94% of them were Italian. According to marital status, 80% of women were married and 20% were single. Regarding the education level, 72% had a high educational qualification, and 28% had a low degree of education. Furthermore, 58% of mothers worked before pregnancy and 57% had some or many economic difficulties (Table 1).

The outcomes detected during the hospital stay of mothers, and at 1-month infant age included the entire sample of women enrolled but, at the 6 months infant age survey, 31.4% of these were lost at follow up. 

During hospitalization, 86% of the mothers breastfed their children, of which 33.7% exclusively, 2.7% predominantly and 49.6% complementary. At the first month, 69% breastfed, of which 37.9% exclusively, 3.1% predominantly and 28% complementary. At the sixth month, 42% of the mothers who responded to the follow-up breastfed only 20.2% exclusively, 7.3% with predominant breastfeeding and 14.5% in a complementary way (Table 2).

The trend of exclusive breastfeeding from the hospital stay (as reference) in the first month had slightly increased (OR: 1.20, CI 95% 1.01–1.43) and it decreased in the sixth month (OR: 0.50, CI 95% 0.40–0.62).

During hospitalization there is a significant association between exclusive breastfeeding and individual deprivation of mothers, with non-linear levels of crude risk of not exclusive breastfeeding in the intermediate classes of deprivation. The same association and trends remain at 1 month and become more important at 6 months. The adjusted OR, obtained with multivariate analysis that used some exposure variables as covariates (pre-birth training, type of birth labor, skin to skin, rooming, prescribed formulated breast milk substitutes, working, twins, age, prematurity and co-sleeping), shows that individual inequalities are not associated with non-exclusive breastfeeding in any of the three detection periods (during hospitalization, at one month and six months) (Table 3).

As for the context inequalities, the crude analysis shows a significant correlation between not following exclusive breastfeeding and the disadvantaged condition deriving from the mothers' residence context only in the highest deprivation classes through the three phases of the study. The adjusted analysis, that used a multivariate model including the same exposure variables of the previous adjusted analysis, highlights no association with exclusive breastfeeding during hospitalization. At the first month there was a growing trend in the weight of inequalities linked to the context, yet without achieving statistical significance. At the 6th months there was a statistical significance association between context inequalities and the risk of not following exclusive breastfeeding in the deprived class (OR: 2.08, CI 95% 1.01–4.27) and in the very deprived class (OR: 1.83, CI 95% 1.00–3.38) (Table 4).

Although in this study some women were lost to follow-up, the sample of mothers referred to at the six months of child age survey was similar for individual inequalities (χ^2^ = 2.99, *p* = 0.559) and contextual (χ^2^ = 0.99, *p* = 0.910) (Table 5), to the sample of mothers enrolled in the first phase. Therefore, it is possible to assert that the estimates of this study at six months are not affected by the possible distortive effects due to a different propensity of mothers to adhere to the follow-up phase.

Therefore, by stratifying context index, the two populations are superimposable, while, considering the individual index there was a modest deviation between the two populations. The 724 women who responded to the six month follow-up showed a better individual socioeconomic status than those who were very poor and represented a lower percentage than those of the total sample.

## 4. Discussion

Breast milk is the gold standard of infant feeding [1,20], nevertheless, the prevalence and duration of breastfeeding have decreased in many countries of the world due to social, economic and cultural reasons [17,21]. Furthermore, the industrialization and the adoption of new lifestyles in many societies have reduced the value of this traditional practice. The significance of breastfeeding has been largely neglected by medical practice, leading to the hypothesis that breast milk can be replaced with artificial products without harmful consequences or, worse, the idea that the latter can bring nutritional and health benefits [17,31]. The initiation and continuation of breastfeeding is linked to several determinants, including social and economic inequalities, the dominant culture in terms of infant feeding, assistance during pregnancy and childbirth, support received, particularly in the early days after childbirth. Knowledge of the influence of the various determinants is important to think of for appropriate intervention programs aimed at promoting, supporting and protecting breastfeeding that involve health services and the various stakeholders in the communities, in order to implement policies to support birth pathways and mothers [32].

The results of this prospective study indicate that during hospitalization, only 33.7% of the sample exclusively breastfed. At the first month, the proportion of mothers who exclusively breastfed (37.9%) grew, but there was a large overall decrease in the proportion of women who were breastfeeding (from 86% in the hospital stay to 69% in one month of life). At the sixth month, only 20.2% of the mothers who responded to the follow-up breastfed exclusively, while 58% did not breastfeed. The prevalence of exclusive breastfeeding measured has been found to be very low in all periods of detection, and this is widely confirmed by regional, national and international studies and surveys [23,24]. In particular, a survey carried out in 2015 at the vaccination centers of the entire Sicilian territory showed that among the mothers presented at the first vaccination access of the child (which in most cases corresponds to the second–third month of life of the child), 30.6% said they were exclusively breastfeeding, 23.1% were breastfeeding in a complementary way and the remaining 46.3% were feeding only with formula [24]. In Italy, according to the Istat data of 2013, the prevalence of exclusive breastfeeding for 4–5-month-old children stands at 38.6% [23].

The multivariate adjusted analysis revealed that individual inequalities do not significantly influence the prevalence of exclusive breastfeeding in any of the three detection periods (hospitalization, one month and six months of child’s life), indicating most likely the central role of good hospital practices. For the context inequalities, the result is different: during the hospitalization, there is no association with exclusive breastfeeding, probably because the hospital plays a "protective" role and reduces context inequalities. However, from the first month, there is a growing trend in the weight of inequalities linked to the context, that lead to a significant association in the most deprived classes at the sixth month. These results indicate that the inequalities in the context begin to emerge from the return home of the mother and the child: if the hospital played a leading role in relation to the exclusive breastfeeding, returning to their own environment and working habits they become determinants of disadvantage of the context, even if Italian law considers some off-hours from work due to breastfeeding practice. It is already marginally evident at one month of a child’s life, and significant at 6 months due to the decreases of the hospital influence and the higher role of the context in which the woman and her own child live. Furthermore, the results at six months can be considered reliable, even if some women have been lost at the follow-up.

These data confirm the results obtained from a survey on SIDS carried out in Sicily on 2692 mothers [24], in which some aspects relating to exclusive breastfeeding were investigated and which revealed an adherence to exclusive breastfeeding associated with the socioeconomic context, being lower among women living in areas of greater disadvantage than women living in the richest areas. Similar results emerged from a study conducted on a sample of more than 4000 mothers from the United States (Florida), in which census data were combined with data relating to the "Fragile Families and Child Well Being Study": the results indicated, in fact, that the neighborhood of residence, unlike the racial or ethnic condition, was strongly associated with behaviors concerning breastfeeding [33]. In particular, mothers who lived in neighborhoods with less disadvantage were more likely to start and support breastfeeding for an adequate time. Moreover, a prospective cohort study carried out in Sweden on 2407 mothers, showed lower prevalence rates of breastfeeding at four months among the infants of populations living in neighborhoods with greater socioeconomic deprivation than those in less deprived neighborhoods [34]. In a survey carried out in Spain, the breastfeeding rates of women living in urban areas were compared with those of women living in rural areas, for a total of 17,067 mothers [35]. Women in urban areas showed greater adherence to the practice of breastfeeding than women in rural areas. Chin and Dozier concluded from their ethnographic research that breastfeeding is a privilege based on social class, regardless of cultural beliefs [36]. Low-income mothers fight and suffer because of the condition of not having adequate income, housing, food, security, healthcare, transportation and dignity. Even for the mother who wants to breastfeed, under these conditions, artificial feeding is almost a forced choice, since the risks and everyday problems that are found in the lives of low-income groups far outweigh the risks of artificial feeding, as all good health practices, breastfeeding is also less practiced in higher inequality classes [37].

In Sicily, many studies have highlighted the impact of disadvantage in morbidity, mortality and cancer incidence in the residents and migrants [38,39,40,41,42]. A study that correlated the levels of inequalities with the use of influenza vaccination and another study that explored the adherence of women to Pap Testing, have confirmed that in Sicily, higher levels of deprivation are related to less use of good health practices [43,44].

Labbok wrote that we need "different paradigm shifts in our perceptions, programs and support for breastfeeding" if we want breastfeeding to become the norm for everyone globally [45]. One of these paradigm shifts is to move from seeing breastfeeding from a medical problem to a sociocultural one. Consistent with this, it is necessary that the protection of breastfeeding, in addition to being focused on value for health, should be considered an element of social justice. This approach could lead to reducing inequalities and injustices based on gender, race and socio-economic status. Everyone must have the right to receive information on the benefits of breastfeeding and the risks associated with artificial feeding, especially the most disadvantaged groups that inherently have difficulty accessing health services and information. The governments have a responsibility to provide this information. Communication channels can play a key role in promoting breastfeeding. The interventions of education and health promotion can, however, if not correctly oriented with suitable tools for the deprived population, have the effect of amplifying the inequalities, rather than reducing them, if not modulated from an equity point of view, that is with a use of resources proportionately higher for the disadvantaged population groups [46].

This is because the likelihood of correctly or entirely understanding a health message (health literacy) is less for people with different or limited cultural tools, or in difficult conditions. The interventions to promote breastfeeding (for example, information material and communication tools aimed at the population, training of health personnel, dissemination of policies, recommendations, guidelines) can contribute to reducing the gap only if well designed and actively offered to women and to disadvantaged groups to a greater extent than to the general population. The same can be said for initiatives to support the mother for breastfeeding (offered by health professionals and peers, already during the period of pregnancy, but especially at birth and after discharge from the maternity ward, until breastfeeding has not stabilized). Conversely, unlike support and promotion interventions, breastfeeding protection initiatives (as the correct implementation of the “International Code on the marketing of breast milk substitutes”, laws, regulations and policies for the protection of working mothers and for the removal of obstacles to breastfeeding outside the home) are less dependent on the transposition by the target population, and contribute to the reduction of inequalities, requiring less corrective actions [46]. As discussed above, the results of our study reveal the importance of the hospital's role in both initiating and maintaining exclusive breastfeeding. It is therefore necessary to implement hospital "best practices" regarding mother- and child-care, according to the ten steps for the success of WHO/UNICEF breastfeeding. They summarize a package of policies and procedures that the facilities that provide maternity and newborn services should be implemented to support breastfeeding and concern the information of pregnant women, the organization of the hospital, the care routines regarding the mother and the child, and the support modalities for breastfeeding after returning home [46]. Mothers need active support during pregnancy and after birth, not only by their families and communities, but also by the entire health system in order to be breastfeeding successfully, both started and established.

## 5. Conclusions

Breastfeeding offers short-term and long-term health, economic and environmental benefits to children, women and society. To realize these advantages, political support and financial investments are needed to protect, promote and support breastfeeding with actions and strategies modulated in an equity perspective.

The results of this study highlight that the risk of not starting or continuing breastfeeding is greater in mothers who have high levels of deprivation and/or live in a low socio-economic context. Therefore, it is important that the prevention policies, already initiated in Sicily, can include all mothers and be strengthened more in the groups of women who have a higher social and cultural disadvantage.

## Figures and Tables

**Table 1:** Socio-demographic characteristics of mothers.

Socio-Demographic Characteristics		N	%
Age classes	18–25	155	15.1
	26–30	280	27.2
	31–35	348	33.8
	>35	247	23.9
Marital status	Married	843	80
	Single/separated/divorced/widow	211	20
Domestic partnership	With the father of the child	1032	98
	With others/alone	21	2
Nationality	Italian	984	94.5
	Foreign	57	5.5
Educational qualification	Low (Elementary/Lower Middle)	294	28
	High (Upper Middle/Degree/Postgraduate)	758	72
Work before pregnancy	No	443	42.2
	Yes	608	57.8
Resources in the end of month	Quite easily/very easily	437	42.6
	With some difficulties/with many difficulties	589	57.4

**Table 2:** Breastfeeding modalities during hospitalization, at one month and at six months.

Breastfeeding Modalities	Hospital Stay		First Month		Sixth Month	
	N	%	N	%	N	%
Exclusive breastfeeding	356	33.7	400	37.9	146	20.2
Predominant	29	2.7	32	3.1	53	7.3
Complementary	523	49.6	295	28	105	14.5
Non-breastfeeding	147	13.9	328	31.1	420	58
Total	1055		1055		724	

**Table 3:** Adjusted risks of non-exclusive breastfeeding and individual inequalities at the end of the three detection times.

SEP	Hospital Stay	First Month	Sixth Month
	OR adjusted * (CI 95%)	OR adjusted * (CI 95%)	OR adjusted * (CI 95%)
2 versus 1	0.78 (0.53–1.15)	0.84 (0.58–1.23)	1.36 (0.83–2.21)
3 versus 1	1.09 (0.69–1.73)	1.26 (0.81–1.96)	1.65 (0.89–3.07)
4 versus 1	0.88 (0.54–1.45)	1.17 (0.73–1.89)	1.89 (0.76–3.04)
5 versus 1	0.71 (0.37–1.37)	0.93 (0.49–1.77)	1.54 (0.52–4.54)

* Odds ratio (OR) adjusted for: pre-birth training, type of birth labor, skin-to-skin, rooming, prescribed formulated breast milk substitutes, working, twins, age, prematurity and co-sleeping. SEP = socio-economic position, CI = confidence interval.

**Table 4:** Adjusted risk of not exclusive breastfeeding and context inequalities at the end of the three detection times.

SEP	Hospital Stay	First Month	Sixth Month
	OR adjusted * (CI 95%)	OR adjusted * (CI 95%)	OR adjusted * (CI 95%)
2 versus 1	1.05 (0.66–1.67)	0.94 (0.60–1.47)	1.26 (0.70–2.27)
3 versus 1	1.49 (0.93–2.39)	1.22 (0.77–1.92)	1.38 (0.76–2.51)
4 versus 1	1.15 (0.70–1.89)	1.19 (0.73–1.92)	2.08 (1.01–4.27) ^
5 versus 1	1.36 (0.86–2.15)	1.51 (0.96–2.36)	1.83 (1.00–3.38) ^

* OR adjusted for: pre-birth training, type of birth labor, skin-to-skin, rooming, prescribed formulated breast milk substitutes, working, twins, age, prematurity and co-sleeping; ^ *p* < 0.05

**Table 5:** Analysis of the different distributions of mothers enrolled at the baseline (during hospitalization and first month) and at the follow-up (six months) by individual and context Socio-economic positions. (χ^2^, *p* < 0.05).

Socio-Economic Position	Individual Socio-Economic Position			Context Socio-Economic Position		
	Baseline Enrolled Mother, N° (%)	Follow up Enrolled Mother, N° (%)	χ^2^	Baseline Enrolled Mother	Follow up Enrolled Mother, N° (%)	χ^2^
1—very rich	77 (7.3)	41 (5.7)	χ^2^ = 2.99 *p* = 0.559	176 (16.7)	121 (16.7)	χ^2^ = 0.99 *p* = 0.910
2—rich	189 (17.9)	125 (17.3)		228 (21.6)	155 (21.4)	
3—medium	210 (19.9)	139 (19.2)		210 (19.9)	152 (21.0)	
4—deprived	344 (32.6)	241 (33.3)		180 (17.1)	112 (15.5)	
5—very deprived	235 (22.3)	178 (24.6)		261 (24.7)	184 (25.4)	
Total	1055	724		1055	724	
